# Level of Insight in Patients With Obsessive–Compulsive Disorder: An Exploratory Comparative Study Between Patients With “Good Insight” and “Poor Insight”

**DOI:** 10.3389/fpsyt.2019.00413

**Published:** 2019-07-03

**Authors:** Richard Chuquel Silveira de Avila, Laura Gratsch do Nascimento, Rafaella Landell de Moura Porto, Leonardo Fontenelle, Eurípedes Constantino Miguel Filho, Vlasios Brakoulias, Ygor Arzeno Ferrão

**Affiliations:** ^1^Programa de Pós-Graduação em Ciências da Saúde, Universidade Federal de Ciências da Saúde de Porto Alegre (UFCSPA), Porto Alegre, Brazil; ^2^Departamento de Psicologia, Universidade Federal de Ciências da Saúde de Porto Alegre (UFCSPA), Porto Alegre, Brazil; ^3^Departamento de Psiquiatria, Federal University of Rio de Janeiro, Rio de Janeiro, Brazil; ^4^Departamento de Psiquiatria, São Paulo University, São Paulo, Brazil; ^5^School of Medicine of Western Sydney University, University of Sydney, Sydney, NSW, Australia

**Keywords:** insight, beliefs, obsessive–compulsive disorder, sensory phenomena, psychopathology

## Abstract

**Introduction:** Insight may be defined as the ability to perceive and evaluate external reality and to separate it from its subjective aspects. It also refers to the ability to self-assess difficulties and personal qualities. Insight may be a predictor of success in the treatment of obsessive–compulsive disorder (OCD), so that individuals with poor insight tend to become refractory to treatment. The objective of this study is to investigate factors associated with poor insight in individuals with OCD.

**Methods:** This cross-sectional exploratory study used the Brown Belief Assessment Scale as a parameter for the creation of the comparison groups: individuals who obtained null scores (zero) composed the group with preserved or good insight (*n* = 148), and those with scores above the 75% percentile composed the group with poor insight (*n* = 124); those with intermediate scores were excluded. Sociodemographic characteristics and clinical and psychopathological aspects, intrinsic and extrinsic to the typical symptoms of OCD, were compared in a univariate analysis. A logistic regression was used to determine which factors associated with critical judgment remained significant.

**Results:** Individuals in the poor insight group differed from those with good insight in regard to: more prevalent use of neuroleptics (*p* = 0.05); higher untreated time interval (*p* < 0.001); higher total Yale–Brown obsessive–compulsive scale score and the obsessions and compulsions factors (all factors with *p* < 0.001); higher dimensional Yale–Brown obsessive–compulsive scale total and dimensional scores (*p* from 0.04 to 0.001); higher prevalence of contamination/cleaning (*p* = 0.006) and hoarding (*p* < 0.001) symptoms dimensions; more prevalent sensory phenomena (*p* = 0.023); higher levels of depression (*p* = 0.007); and more prevalent comorbidity with bipolar affective disorder (*p* = 0.05) and post-traumatic stress disorder (PTSD) (*p* = 0.04). After analyzing the logistic regression, we conclude that the most important factors associated with poor insight are: the presence of any sensory phenomena (OR: 2.24), use of neuroleptics (OR: 1.66), and hoarding symptoms (OR: 1.15).

**Conclusion:** The variability of insight in patients with OCD seems to be an important psychopathological characteristic in the differentiation of possible subtypes of OCD, since the poor insight is associated with sensory phenomena and greater use of neuroleptics, which makes it possible to conjecture the role of dopaminergic neurocircuits in the neurobiology of this disorder. In addition, there is also an association with the symptoms of hoarding content, admittedly one of the symptomatic contents with less response to conventional OCD treatments. Studies based on neurobiological aspects such as neuroimaging and neuropsychology may help to elucidate more consistently the role of insight in patients with OCD and the repercussions concerning available treatments.

## Introduction

“Poor insight,” or the deficit of the capacity of judgment, is usually associated with intellectual cognitive poverty, and it may decrease the capacity of evaluation of the reality despite evidence to the contrary (1). The process may be similar to that in delirium (2, 3), overvalued ideas (4), obsessions (5), or even in regular beliefs or automatic thoughts in people without a psychiatric diagnosis (6). Classically, the term “insight” is used in psychoanalysis to illustrate, in the therapeutic environment, the sudden understanding of something or some situation, which involves, in a certain way, the capacity to learn something. “Insight” can also be defined as the convergence of several judgments that lead the individual to the conclusion of a problem by non-means (7, 8), or “a form of evaluation and perception of internal power” or “a capacity for selection and prediction of consequences” (9, 10). Its function is self-evaluation, as it is able to measure difficulties and qualities (11). “Poor insight” means not understanding, perhaps even questioning, what is being done in a given situation (whether right or wrong, if appropriate or not). According to David, the concept of insight comprises three components characterized by: 1) recognition of the disease itself, 2) the ability to recognize symptoms, and 3) compliance with treatment. It is a transdiagnostic concept, applicable to many psychiatric disorders (12).

There is extensive literature on insight in patients with psychotic disorders, such as schizophrenia, delusional disorders, bipolar disorder, suicidal behavior, and neurological conditions (13–21), specially neuroimaging studies that show correlation of insight level and some brain structures as: dorsal precentral and postcentral gyri, dorsal frontal and parietal cortices (22), and ventrolateral prefrontal cortex (23), which allow us to conjecture the possibility of a neurobiological constituent for insight, especially a network of frontal, temporal, and parietal brain regions (23–25), including posterior insula as a main network node (26).

Obsessive–compulsive disorder (OCD), on the other hand, is characterized by obsessions (thoughts, images, or intrusive impulses that cause emotional discomfort) and compulsions (behaviors performed to diminish or deal with the discomfort created by obsessions) (27, 28). The OCD patient is classically considered to have a good level of insight regarding their symptoms. The OCD patient, in general, understands their symptoms as ego-dystonic, that is, impulses, wishes, or thoughts that are unacceptable or repugnant to the ego or self (29), leading patients to realize that the obsession is totally contrary to the patients’ wishes and desires. Therefore, people with OCD are aware that their behaviors are abnormal and responding to their compulsions causes them anxiety and distress. It is very common, meanwhile, that at the exact time of the obsession/compulsion occurrence, patients present an oscillatory conviction (doubt) about the nature (true or false) of the obsession, resulting again in anxiety and distress (30). Thus, patients with OCD may present diverse psychopathological features regarding levels of insight, ego dystonicity and conviction about their own symptoms. The similarity, inconsistency, complexity, and/or overlapping of the cited conceptual constructs (and others, as “beliefs,” “overvalued ideas,” and even “delusional thoughts”) (8, 31–34) have led researchers to confound the cited concepts and to use these terms very loosely, since adequate instruments to assess them are not often used.

Therefore, the gap in the knowledge of the concept and the influence of insight on patients with OCD have been generating efforts to understand and to measure this psychopathological construct, resulting in the fact that its role in psychiatric disorders is being increasingly recognized. Recently, the *Diagnostic and Statistical Manual of Mental Disorders, 5th edition* (27) included two specifiers for OCD diagnosis: the presence of tics and, precisely, the level of insight, which may be classified in “good or fair” insight, “poor insight.” and “absent” insight/delusional beliefs. Insight in this context refers to construct regarding the reasonableness of one’s belief, not in relation to whether one believes that they have OCD, or whether they believe in receiving treatment.

To better highlight the importance of the topic (and also to illustrate the heterogeneity of how it has been approached), 4% to 36% of OCD patients have poor or no insight about their symptoms and their pathology (8, 35–38). This poor insight subset of patients can accommodate their symptoms and take more time to seek treatment, which would associate poor insight with longer duration of illness or longer time without treatment, all of which are negative predictors for the therapeutic process of OCD, showing a worse prognosis (8, 35, 39–46).

Thus, the investigation of factors associated with poor insight in patients with OCD may help to understand some psychopathological and neurobiological aspects and to predict the response to the current conventional treatments. Therefore, the objective of this exploratory study is to verify the association of the level of insight (to a greater or lesser degree) with a great number of clinical variables in patients with OCD. Not only the presence, but also the severity of the obsessive–compulsive symptoms (OCS) content dimensions according to the dimensional Yale–Brown obsessive–compulsive scale (DY-BOCS) and sensory phenomena were evaluated. Instead of using only the Yale–Brown obsessive–compulsive scale (Y-BOCS) (45) item for insight, we assessed the presence and severity of insight with a specific instrument: the Brown assessment beliefs scale (BABS) (32). Thereby, according to the literature and to our clinical experience, we hypothesize that OCD patients with poor insight will present: earlier age of onset of obsessive–compulsive symptoms (OCS) (47, 48), longer duration of illness (8, 39, 48), higher prevalence of familial history of OCD (49), higher prevalence of neuroleptics prescription (50), higher prevalence of suicidality, more common presence and higher severity of specific OCS content [especially for contamination/washing/cleaning (51) and hoarding (36, 47, 52)], higher prevalence of any sensory phenomena (53), higher severity of depressive (54) and anxious symptoms, and higher prevalence of specific comorbid psychiatric disorders [especially major depression (47, 48), dysthymia (55), bipolar disorder (56), and delusional disorder (57)].

## Materials and methods

This is a cross-sectional study with the 1,001 patients database of the Brazilian Consortium for Research on Obsessive–Compulsive Spectrum Disorders (CTOC) (58), which collected data between 2003 and 2008 in seven research centers in three different Brazilian regions. The inclusion criteria for the study were: being in treatment at one of the research centers, fulfilled criteria for OCD according to the *Diagnostic and Statistical Manual of Mental Disorders, 4th Edition* (DSM-IV) and confirmed by structured clinical interview, and being able to understand and participate in the research protocol. Comorbid diagnosis of schizophrenia was excluded. The sources of recruitment of the participants were public and private psychiatric outpatient and inpatients services. Further methodological detail (as who have performed the assessment and interrater reliability) is described in Miguel et al. (58). The present study was submitted and approved by the ethics committees of the centers previously involved (USP-968/05; IPA-6600023; UFRGS-06/171; Unifesp-302/2006).

### Instruments

- *Sociodemographic and clinical questionnaire of the Brazilian Consortium for Research on Obsessive–Compulsive Spectrum Disorders (CTOC):* This questionnaire was created by the CTOC researchers as an initial instrument in which sociodemographic, socioeconomic data, medical history data, and a semi-structured interview on family psychiatric history were used to characterize each individual. Therefore, basic information was collected: age, weight, height, economic class, naturalness, marital status, number of children, origin, number of people living with, religion, education, others. A general medical history is also questioned in addition to the psychiatric history, which details the medications already used and the resulting effects, as well as variables related to suicidality (ideation, plan, attempt, hospitalization due to, and familial history) and other treatments, such as psychotherapy.
- *Structured clinical interview for DSM-IV axis I disorders, research version:* DSM-IV structured interview for personality disorders. Not yet validated for DSM 5 in Brazilian Portuguese. These semi-structured interviews were used as a screening tool to assess the presence or not of OCD and psychiatric comorbidities (59).
- *Yale–Brown obsessive–compulsive symptom scale (Y-BOCS):* This scale has 10 items, five for obsession evaluation and five for compulsion assessment. Each item can be answered by the individual on a scale of 0 (nonlinear or less pathological) to 4 (delirious or more pathological). This scale provides three scores: severity of obsessions, severity of compulsions, and total severity of OCD (45).
- *Dimensional scale for the assessment of the presence and severity of obsessive–compulsive symptoms (DY-BOCS):* This scale is composed of six subscales according to the content of OCS (1—aggression, violence, and natural disasters; 2—sexual and religious; 3—symmetry, order, counting, and arrangement; 4—contamination, cleaning, and washing; 5—accumulation; and 6—miscellaneous content), also generating a total score of symptoms. It also has a list of symptoms (88 items), for each of the abovementioned thematic dimensions (60). The Miscellanea dimension was not evaluated in this work because it is a very heterogeneous dimension, with symptoms such as concern for diseases, magic numbers, among others, which did not add to the other factors in factorial analysis.
- *Sensory phenomena scale of the University of São Paulo:* sensorial phenomena are sensory experiences or subjective sensations, such as feelings of incompleteness, urgency, or “having to do until you feel it is right,” that precede repetitive behaviors such as tics or compulsions (61). They do not obey the logic of an obsessive thought, because they are not cognitive phenomena, but can generate anxiety and can be neutralized by behaviors, gestures, or tics. This scale was created to measure the severity of sensory phenomena occurring before or during the performance of repetitive behaviors. This scale is divided into two parts. The first is a list of the presence of current and past phenomena. The second is a severity scale that evaluates four aspects: frequency, amount of suffering, interference in the patient’s life, and a total score of the three previous ones (62).
- *Beck depression inventory (BDI):* This inventory was created by Beck and colleagues in 1961 (63) and is intended to assess the severity of depressive symptoms. It is a self-report instrument composed of 21 items with four response options for each item with values ranging from 0 to 3 (64).
- *Beck’s anxiety inventory (BAI):* Like BDI, BAI has 21 items with four response options related to anxiety intensity within a 1-week period (65).
- *Brown belief assessment scale (BABS):* This scale was constructed to rate (rather than using a dichotomous model of “with or without insight”) the degree of conviction and insight that patients have concerning their beliefs. These beliefs include delusions as well as the beliefs that may underlie obsessional thinking or phobias. For patients diagnosed with OCD, it is recommended that the obsession that generated the most concerns in the previous week is considered as the “belief” and then respond to six dimensions that can be scored from 0 (absence of pathology) to 4 (delusional or more pathological). The dimensions of insight are: conviction, perception of others’ view of belief, explanation of different views, rigidity of ideas, attempt to refute ideas, and ability of insight. The seventh dimension is not part of the total score: ideas/delusions of reference (32). For the purposes of this work, this instrument served to obtain the comparison groups. According to the scores of the total sample (*n* = 968; 33 were missing), two groups were formed: one with the preserved “good” insight (GI group) (*n* = 148; 15.3%), whose final BABS score was “zero” (that is, perfectly preserved insight) and another with the poor insight (PI group) (*n* = 124; 12.8%), whose final BABS score was above the 75% percentile (score ≥ 14). Thus, 696 (71.9%) patients with BABS score between 1 and 13 were excluded. A whole sample study was already published (36), when instead of 1.001, it was possible to proceed the analysis with 842 patients. At that study, two variables were related to poor insight: hoarding and overall OCD severity. Thus, we decided for another strategy that could allow us to better explore the phenomenological aspects of poor insight in OCD patients. The selective sampling of phenotypically extreme individuals’ strategy has been widely used to increase power when comparing some clinical or genetic features (66).

### Statistics

Continuous variables were described as mean (standard deviation) when they had normal distribution and as median (minimum–maximum) when there was no normal distribution. Categorical variables were described as absolute values (*n*) and relative values (%). Normal distribution was assessed by the Kolmogorov–Smirnov test. The means were compared by the Student’s *t*-test and the medians by the Mann–Whitney *U*-test. Pearson’s chi-square, Yates, or Fisher’s exact test were used to compare categorical variables between the two groups. Variables with a *p*-value ≤ 0.10 in the univariate analysis, respecting multi-collinearity and clinical-epidemiological relevance, were included in a backward binary multiple logistic regression model of Wald to determine factors independently associated with the level of insight. The regression model included all the variables that were significant in the univariate analyses, except those with variance inflation factor <1 or >5, which means high collinearity (especially for continuous variables). After the logistic regression, only variables with a *p*-value ≤ 0.05 were selected to remain in the model, since it is a conservative way to control multiple comparisons. The odds ratio, with 95% confidence intervals was also calculated for the remaining forms in the regression model. SPSS software 22.0 and WinPEPI 11.0 were used to perform the analysis.

## Results

### Sociodemographic and Clinical Data

The sociodemographic and clinical aspects of the subjects of both groups are compared in **Table 1**. There is a tendency the good insight group to frequently have more individuals in the class A (*p* = 0.07). Otherwise, the group with poor insight showed a more prevalent current use of neuroleptics (*p* = 0.05).

**Table 1:** Sociodemographic and clinical data: comparison between patients with poor and good insight.

	Poor insight (*n* = 124); BABS ≥ 14		Good insight (*n* = 148); BABS = 0		Statistical test	*p*
	Median	(Min–Max)	Median	(Min–Max)		
Age	35.5	(13–68)	32	(10–77)	U_MW_ = 8,205.0	0.13
Studied years	14	(2–31)	14	(1–25)	U_MW_ = 8,412.0	0.27
	***n***	**%**	***n***	**%**		
Male gender	52	41.9	72	48.6	X^2^_Yates_ = 0.97	0.33
No spouse	76	61.3	79	53.4	X^2^_Yates_ = 1.56	0.21
No occupation	27	18.2	23	18.5	X^2^_Yates_ < 0.01	1.00
Ethnicity: White	94	75.8	121	81.8	X^2^_Yates_ = 1.11	0.29
Socioeconomic Classification Class A Class B Class C Class D Class E	8 54 48 11 3	6.5 43.5 38.7 8.9 2.4	26 58 54 7 3	17.6 39.2 36.5 4.7 2.0	X^2^_Pearson_ = 8.87	0.07
Current treatments						
Pharmacological —SSRIs —Other antidepressants —Benzodiazepines —Mood stabilizers —Lithium —Neuroleptics	91 15 57 16 8 35	73.4 12.1 46.0 12.9 6.5 28.2	113 20 61 18 6 26	76.4 13.5 41.2 12.2 4.1 17.6	X^2^_Yates_ = 0.18 X^2^_Yates_ = 0.03 X^2^_Yates_ = 0.44 X^2^_Yates_ < 0.01 X^2^_Yates_ = 0.38 X^2^_Yates_ = 3.81	0.67 0.87 0.51 1.00 0.54 0.05
Any psychotherapy CBT	73 20	58,9 27,4	96 25	64,9 26,0	X^2^_Yates_ = 0,79 X^2^_Yates_ = 0,02	0.37 0.90
Internment	15	12,1	8	5,4	X^2^_Yates_ = 0,47	0.49

p, level of statistical significance; n, absolute value; %, relative value; Min, minimum; Max, maximum; UMW, Mann–Whitney U test; χ^2^_Yates_, Yates chi-square test; χ^2^_Pearson_, Pearson chi-square test; SSRI, selective serotonin reuptake inhibitor; CBT, cognitive-behavioral therapy; BABS, Brown Belief Assessment Scale.

Regarding the BABS dimensions descriptive results, the OCD patients with poor insight (total score ≥ 14) presented the following median (minimum–maximum) scores: conviction of the belief: 4 (1–4); perception of others’ view of belief: 1 (0–4); explanation of the differing views: 3 (0–4); fixity of beliefs: 3 (0–4); attempt to disprove beliefs: 3 (0–4); ability of insight: 2 (0–4); and ideas/delusions of reference: 0 (0–4). Regarding the maximum score of each dimension, 81 (65.3%) of the patients are totally convinced about the reality of the “beliefs” (obsessions); 13 (10.5%) think that other people also believe completely in the “beliefs” (obsessions); 45 (36.3%) of the patients still believe in the “beliefs” (obsessions) even with the disagreement of other people about that; 44 (35.5%) completely rejected “beliefs” could be false; 78 (62.9%) makes no attempt to refute the “beliefs;” 33 (26.6%) believe that “beliefs” are not of a psychiatric nature; and 9 (7.3%) scored ideas/delusions of reference.

### Level of Insight and the Psychopathological Intrinsic Phenomena of OCD

Some clinical characteristics of OCD were different for individuals with poor insight when compared with those with good insight (**Table 2**): the first group started the treatment later (*p* < 0.001) and remained longer untreated (*p* < 0.001); showed greater overall severity of symptoms in both Y-BOCS and DY-BOCS; obtained greater severity scores in any of the symptomatologic dimensions of the DY-BOCS; higher prevalence of symptoms of symmetry, contamination/cleaning, and hoarding, according to the list of symptoms of DY-BOCS; and higher prevalence of sensory phenomena.

**Table 2:** Intrinsic phenomenological features of obsessive–compulsive disorder (OCD): comparison between patients with poor and good insight.

	Poor insight (*n* = 124); BABS ≥ 14		Good insight (*n* = 148); BABS = 0		Statistical test	*p*
	Median	(Min–Max)	Median	(Min–Max)		
OCS age of onset	10	(3–37)	11	(4–43)	U_MW_ = 7,680.0	0.17
Period without treatment	17	(0–56)	11	(0–58)	U_MW_ = 5,281.0	**<0.001**
YBOCS —Total Score —Obsessions —Compulsions	29 14 15	(8–40) (4–20) (4–20)	23 11 12	(7–37) (1–18) (2–20)	U_MW_ = 4,670.0 U_MW_ = 4,651.0 U_MW_ = 5,286.0	**<0.001** **<0.001** **<0.001**
D-YBOCS severity —Total Score —Aggressiveness —Sex/Religion —Symmetry/Ordering —Contamination/Cleaning —Hoarding	24 7 6 9 9 3.5	(4–30) (0–15) (0–15) (0–15) (0–15) (0–15)	21 3 0 6 3.5 0	(0–30) (0–15) (0–15) (0–15) (0–14) (0–13)	U_MW_ = 6,476.5 U_MW_ = 7,220.0 U_MW_ = 7,599.0 U_MW_ = 6,730.5 U_MW_ = 6,676.5 U_MW_ = 6,051.0	**<0.001** **0.04** **0.01** **<0.001** **<0.001** **<0.001**
Severity Sensory Phenomena*	9	(1–15)	9	(1–15)	U_MW_ = 3,513.5	0.24
	*n*	*%*	*n*	*%*		
Family History of OCD	60	48.4	71	48.0	X^2^_Yates_ < 0.001	1.00
Family History of Tics	25	20.8	22	15.7	X^2^_Yates_ = 0.82	0.37
D-YBOCS dimensions —Aggressiveness —Sex/Religion —Symmetry —Contamination/Cleaning —Hoarding	87 77 113 98 83	70.2 62.1 91.1 79.0 66.9	95 83 121 93 59	64.2 56.1 82.3 62.8 39.9	X^2^_Yates_ = 0.83 X^2^_Yates_ = 0.78 X^2^_Yates_ = 3.71 X^2^_Yates_ = 7.71 X^2^_Yates_ = 18.75	0.36 0.38 **0.054** **0.006** **<0.001**
Presence of any Sensory Phenomena	94	75.8	92	62.2	X^2^_Yates_ = 5.20	**0.023**

*Poor insight (n = 92); Good insight (n = 85); p, level of statistical significance; n, absolute value; %, relative value; Min, minimum; Max, maximum; UMW, Mann–Whitney U test; χ^2^_Yates_, Yates chi-square test; SOC, obsessive compulsive symptoms; YBOCS, Yale–Brown Obsessive-Compulsive Symptom Scale; DYBOCS, dimensional scale for the assessment of the presence and severity of obsessive-compulsive symptoms; BABS, Brown Belief Assessment Scale.

### Level of Insight and the Psychopathological Extrinsic Phenomena of OCD

The depression scores, measured by BDI, were higher in the group with poor insight (*p* = 0.007). A statistical trend to higher anxiety levels according to the BAI was shown for the poor insight group (*p* = 0.096). Among the psychiatric comorbidities that were associated with the poor insight group, major depression disorder (*p* = 0.08) and attention deficit hyperactivity disorder (*p* = 0.07) showed statistical tendency to the association, while bipolar disorder (*p* = 0.05) and simple phobia (*p* = 0.04) showed a relevant association. Neither suicidality nor other psychiatric comorbidities were significantly related to poor insight (**Table 3**).

**Table 3:** Extrinsic phenomenological features of OCD: comparison between patients with poor and good insight.

	Poor insight (*n* = 124); BABS ≥ 14		Good insight (*n* = 148); BABS = 0		Statistical test	*p*
	Median	(Min–Max)	Median	(Min–Max)		
BDI	17	(0–52)	13	(0–53)	U_MW_ = 7,384.5	**0.007**
BAI	15	(0–51)	12	(0–48)	U_MW_ = 8,045.0	**0.096**
	*n*	%	*n*	%		
Suicidality —Ideation —Plan —Attempt —Hospital Internment —Familial History	48 25 11 4 23	40.3 21.0 9.2 3.2 19.3	55 29 15 4 22	39.3 20.7 10.7 2.7 15.7	X^2^_Yates_ = 0.002 X^2^_Yates_ < 0.001 X^2^_Yates_ = 0.03 X^2^_Yates_ = 0.65 X^2^_Yates_ = 0.36	0.96 1.00 0.85 0.80 0.55
Comorbidities —Major Depression —Dysthymia —BAD —Delusional Disorder —Anxiety Disorders —Panic —Agoraphobia —Social Phobia —Simple Phobia —PTSD —GAD —Alcohol Abuse/Dependence —Tics —Tourette Disorder —Separation Anxiety —ADHD	51 16 6 6 10 3 32 30 21 32 5 34 10 7 22	41.1 12.9 4.8 4.8 8.1 2.4 25.8 24.2 16.9 25.8 4.0 27.4 8.1 5.6 17.7	42 21 1 2 16 7 35 36 12 49 6 41 9 6 14	28.4 14.2 0.7 1.4 10.8 4.7 23.6 24.3 8.1 33.1 4.1 27.7 6.1 4.1 9.5	X^2^_Yates_ = 4.94 X^2^_Yates_ = 0.02 Fisher Fisher X^2^_Yates_ = 0.31 Fisher X^2^_Yates_= 0.08 X^2^_Yates_ < 0.001 X^2^_Yates_ = 4.14 X^2^_Yates_ =1.39 X^2^_Yates_ =1.20 X^2^_Yates_ < 0.001 X^2^_Yates_ = 0.16 X^2^_Yates_ = 0.11 X^2^_Yates_ = 3.34	**0.08** 0.90 **0.05** 0.15 0.58 0.35 0.79 1.00 **0.04** 0.24 0.55 1.00 0.69 0.75 **0.07**

p, level of statistical significance; n, absolute value; %, relative value; Min, minimum; Max, maximum; UMW, Mann–Whitney U test; χ^2^_Yates_, Yates chi-square test; BDI, Beck Depression Inventory; BAI, Beck Anxiety Inventory; BAD, bipolar affective disorder; PTSD, posttraumatic stress disorder; GAD, generalized anxiety disorder; ADHD, attention deficit hyperactivity disorder; BABS, Brown Belief Assessment Scale.

### Logistic Regression Analysis

**Table 4** shows the logistic regression results. The variables that were included in the model were: socioeconomic class; current use of neuroleptics; time interval without treatment; presence of symptoms of symmetry, contamination/cleaning, hoarding according to the DY-BOCS; Y-BOCS obsessions and compulsions scores; all factors related to severity as measured by the DY-BOCS; BDI and BAI total scores; comorbidity with major depression, bipolar disorder, simple phobia, and attention deficit hyperactivity disorder. The total Y-BOCS score was excluded from the regression analysis by having variance inflation factor = 11.56 (collinearity with Y-BOCS obsessions score). “Considering that the Y-BOCS total score includes the sub score of compulsions, which is a behavioral phenomenon, and as poor insight and obsessions are both cognitive phenomena, the obsessions score was considered more clinically relevant for the purposes of this study.” Thus, the presence of sensory phenomena (OR = 2.24) remained in the model with statistical significance. Other statistical relevant variables remained in the model: current use of neuroleptics (OR = 1.66); total score of the dimensional Yale–Brown obsessive-compulsive scale (DY-BOCS) hoarding dimension (OR = 1.15); and the time interval without treatment (OR = 1.05).

**Table 4:** Binary logistic regression results (WALD backwards) with predictor variables (*p* ≤ 0.10) of the univariate analysis.

	Wald χ^2^	*p*	OR	CI 95% of OR
Current use of Neuroleptics	8.88	0.003	1.66	1.31–1.84
Period without treatment	13.61	<0.001	1.05	1.02–1.08
Severity of Hoarding dimension	14.22	<0.001	1.15	1.07–1.24
Presence of any sensory phenomena	6.20	0.013	2.24	1.19–4.22
Variable inserted in the steps of Regression: step 1—Gravity of the accumulation dimension; step 2—Time without treatment; step 3—Current use of neuroleptics; step 4—Presence of some sensory phenomenon.				

Wald χ^2^, Wald chi-square test; p, level of statistical significance; OR, odds ratio; CI, confidence interval.

## Discussion

Although many variables were statistically significant in the univariate analysis, only four remained significantly associated with the presence of poor insight in patients with OCD after logistic regression analysis. However, one of them (time without treatment), despite the statistical significance, did not obtain clinical-epidemiological relevance (OR = 1.05), leading us to discuss the remaining variables in the regression model: the presence of any sensory phenomena, the use of neuroleptics, and severity of the DY-BOCS hoarding dimension.

### Sensory Phenomena and Poor Insight in Patients with OCD

Among the results, the most significant was the relation of the sensory phenomena with poor insight (OR = 2.24). The relation of the low insight capacity with sensory phenomena was previously reported by Ferrão et al., who, when comparing patients with OCD with and without sensory phenomena, found higher BABS scores for the first group (median equal to 7 and 5, respectively, *p* = 0.007) (4). As suggested by some authors (67–70), the presence of sensory phenomena may predict treatment failure, which can be understood not only by its possible specific neurobiological aspects, but also by the co-occurrence of poor insight (71, 72), which can itself reduce treatment engagement and reduce the chances of an appropriate treatment response. If for psychopharmacological approaches this statement is valid, the presence of sensory phenomena and poor insight leads also to increased rates of nonresponse in psychotherapeutic therapies (39). Moritz et al. reported that the presence of sensory phenomena in patients diagnosed with OCD predicts poor insight, but it depends on the type of sensory phenomenon, with special emphasis on visual and tactile phenomena (not analyzed in our study) (71). In this study, a positive association was also found between the severity of the sensory phenomenon and the severity of the compulsions, which may be because the sensory properties may increase the subjective reality of obsession that hinders the task of discarding thought and, thus, turning compulsions more difficult to resist (71). Consequently, sensory phenomena (and a lower insight about symptoms) would reinforce the “vicious cycle” of OCD.

Some data suggest that patients with sensory phenomena consider this trigger more important than obsessions and consequently have stronger compulsions (62, 73). About 65% of patients with OCD perform compulsions due to sensory phenomena (62). The data above corroborate a previous study, in which poor insight about the symptoms allows the individual to have a smaller capacity to interrupt or control the OCS (36), perceiving them as “correct,” “adequate,” “relevant,” or even “necessary.”

A neurobiological explanation for this precise association is not yet possible, but some conjectures may be done. It is known that in OCD patients, the anterior dorsolateral, orbitofrontal, and anterior cingulate prefrontal cortex are usually hyperactive. In general, these structures are involved in intersections with other neurocircuits to retain information, manipulate options, and assist in decision making and goal maintenance (74). When they connect with limbic circuits, for instance, they add emotional aspects (rewards) to information and decision making and provide the most diverse forms of behavior (75). The neurobiological dimensional approach recently proposed by the National Institute of Mental Health (76, 77) named Research Domain Criteria (https://www.nimh.nih.gov/research-priorities/rdoc/index.shtml) describes that the default mode network, which basically involves brain structures such as medial anterior prefrontal, posterior cingulate cortex, and angular gyrus, is hyperactive in patients with OCD (78, 79). This circuit, in healthy people, becomes more active at rest or when people are asked to reflect on their own thoughts. Thus, obsessions (intrusive thoughts) could be a consequence of the hyperactivity of this circuit (78), and this “hyperactivation” can be stimulated or facilitated by intersections with structures of other neurocircuits. One such structure is the insula, which together with the anterior cingulate cortex and the amygdala, constitute another neural circuit known as a “protrusion circuit,” which detects any “protrusion” or aspects that stand out in our perception in the environment (both external and interoceptive aspects) (79, 80). Thus, it can be postulated that in patients with OCD, the “negative valence systems” (primarily responsible for responses to aversive situations or context, such as fear, anxiety, and loss) could, for some reason, be also hyperactive, increasing the frequency and intensity of the perception of internal (interoceptive) sensations, becoming what we call “sensory phenomena.” Once the “protrusion circuit” has been activated through insular connections (78), there would be also hyperactivation of the default network circuit and, thus, the increase or maintenance of OCD symptoms. The insula is also responsible for connecting the two circuits above with other two circuits known to be involved in OCD (81, 82): positive affects (responsible for the sensitivity to the presence of protrusions in the external or internal environment—this circuit involves the basal ganglia) and the cognitive (which is responsible for procedural memory and selective attention—this circuit involves, for example, the dorsolateral prefrontal cortex) (79). The direction of the influences of one circuit in the other (and vice versa), however, is still a point of discussion, but it seems little evident (from clinical experience) that the motor circuits (which generate the compulsions) can generate hyperactivation of salience circuits, causing sensory phenomena; the opposite seems more likely to happen. Thus, the association of sensory phenomena with OCD with poor insight may be based on intersections of neurocircuits with distinct functions but that interact to manifest heterogeneously what we call OCD.

### Use of Neuroleptics and Poor Insight in Patients With OCD

Neuroleptics are not the first-choice treatment for OCD, but it seems to be valid as adjuvant when treating resistant or refractory OCD (83), especially atypical neuroleptics, which have augmenting synergism with selective serotonin reuptake inhibitors (SSRIs) because they also have serotonergic action (83, 84). As the CTOC sample is predominantly composed of specialized and tertiary health services, the recruitment of more severe patients, nonresponders to conventional and complex treatments (with comorbidities with tics, for example), may have biased our results, leading to a greater prevalence of the use of these specific medications in these centers (39, 42, 85). As poor insight was related to sensory phenomena, and since sensory phenomena are more prevalent in patients with OCD who also have tics (64, 86), we could speculate a “dopaminergic” modulation of “poor insight.” It could be explained by the facts that tics occur due to dopaminergic dysfunctions involving the basal nuclei, especially striatum and substantia nigra (87, 88), which may result clinically in the increased prescription of drugs with dopaminergic action, such as neuroleptics. Thus, the association of an atypical neuroleptic with SSRIs could act, in these cases, with synergism by serotonergic potentiation or with synergism by addition, adding effect in the involved dopaminergic neurocircuits. Another possibility to justify the association of poor insight and neuroleptics is the fact that, in certain patients with OCD, poor insight may, depending on its severity, remind clinical practitioners of delusion or even psychotic functioning (89, 90), leading psychiatrists to prescribe neuroleptics in association with SSRIs (35, 83, 91, 92).

### Severity of the DY-BOCS Hoarding Dimension and Poor Insight in Patients With OCD

According to the regression performed in this study, the greater severity of the DY-BOCS hoarding dimension was associated with poor insight. Similar results are pointed out in the literature when correlating a worse insight capacity with hoarding symptoms (47, 48, 51, 93–97). The reason so many papers agree to this association still needs further study. Kalogeraki and Michopoulos (97) suggest a cognitive model for hoarding disorder that includes four factors: 1) personal vulnerability, including aspects, such as heredity, stressful life events, personality traits, and interpersonal difficulties; 2) difficulties in information processing, such as attention deficit, memory and executive functions, with difficulty to make decisions and categorize; 3) dysfunctional cognitive content, such as ownership, emotional attachment to possessions, dysfunctional beliefs about mnemonic ability, and the importance of memories; and 4) hoarding behaviors and their positive and negative reinforcement, such as pleasure in acquiring/keeping or anxiety/discomfort to discard (97). Failure to be critical in relation to hoarding acts (associated or not to the diagnosis of OCD) may be a consequence of the sum of cognitive dysfunctions of more than one of these factors. In this sense, both the neural circuits associated with the greater significance of rewards for possession/accumulation (circuits of positive valence systems, primarily responsible for responses to positive motivational situations or contexts, such as reward seeking, consummatory behavior, and reward/habit learning) and greater aversion to frustration by discard/insecurity (circuit of negative valence systems) may be hyperactive simultaneously (79). Of course, the association of the severity of the hoarding symptoms would reflect a higher intensity or complexity of interaction of these circuits, leading to a more committed insight, specifically about this symptomatology.

## Conclusions

This cross-sectional exploratory study was conducted in seven tertiary research centers in three different Brazilian regions, which may alert us to interpret the results with caution, since the generalization of the results to all sort of OCD patients is limited. Due to the methodology and recruitment strategy, it may be argued that only moderate to severe patients answered the questionnaires and, thus, results may be related only for those cases. Moreover, the sample sizes were small and not equal, which may had led to loss of statistical efficiency. Nevertheless, interesting results have been found and deserve attention.

Our results showed that patients with OCD with poor insight seem to present some specificities such as: higher presence of any sensory phenomena, higher prevalence of neuroleptic use, and greater severity of hoarding symptoms. Although the methodological nature of the study does not allow causal inferences, we can conjecture that: 1) sensorial phenomena and severity of hoarding symptoms lead to a poorer insight; and 2) a poorer insight leads psychiatrists to use neuroleptics more frequently. Neurobiological and pathophysiological aspects, as well as reactive cognitive dysfunctions, may justify the first statement, while empirical observations lead to an evidence-based clinical practice that justifies the second statement in some situations. The more detailed exploration from the psychopathological and neurobiological point of view of the sensory phenomena and their subtypes in patients with OCD could help in the better understanding of how these phenomena would make it difficult for the patient to perceive the pathological nature of the symptoms. This study did not evaluate whether the use of neuroleptics in patients with OCD with poor insight had adequate response, which could be answered only in specifically designed prospective or clinical trials. Therefore, intervention studies in this subpopulation, whether with psychotropic drugs, psychotherapeutic techniques, or neurobiological therapies, should be stimulated and conducted properly. Because of the heterogeneity of OCD, the more detailed understanding of insight in patients with OCD should include in future studies the application of instruments that assess this phenomenon for each of the dimensions of DY-BOCS, in other words, how well the patient can judge as reasonable the contents of each of the symptoms dimensions. These results point to the need to explore patients with OCD with a poor insight, since they constitute a special and not uncommon (prevalence of 12.8% among OCD patients) subtype of patients who may require a greater effort by health professionals and services, mainly due to its greater complexity and the difficulty to respond to conventional available treatments.

## Data Availability Statement

The datasets generated for this study are available on request to the corresponding author.

## Ethics Statement

This study was carried out in accordance with the recommendations of 196/96 Resolution, from the National Commission on Research Ethics, from the National Health Council of the Brazilian Health Ministry with written informed consent from all subjects. All subjects gave written informed consent in accordance with the Declaration of Helsinki. The protocol was approved by imvolved local ethics committees (USP, UNIFESP, UNESP, UFRJ, UFRGS-IPA, UFBA, UPE).

## Author Contributions

RA, LN, and RP wrote the manuscript and contributed with review and the main insights in data analysis and discussion. LF, EF and YF have planned the dataset of the consortium, coordinated the data collection, and reviewed the insights in data analysis and discussion. VB proposed some change in the basic conceptual aspects of the paper and reviewed it carefully, concerning data analysis and discussion. YF performed the statistical analysis, guided the preparation, and reviewed the manuscript.

## Funding

This study was financed in part by the Coordenação de Aperfeiçoamento de Pessoal de Nível Superior - Brasil (CAPES) - Finance Code 001, especifically for Leonardo F. Fontenelle, Eurípedes Constantino Miguel Filho and Ygor Arzeno Ferrão.

## Conflict of Interest Statement

The authors declare no conflict of interest for the purpose of this study. The present work was carried out with the support of the Coordination of Improvement of Higher Education Personnel—Brazil (CAPES)—Financing Code 001 [YF, LF, and EM are stockholders of the National Research Council (CNPq); RC and LN are CNPQ scientific initiation fellows].
